# CNS inflammatory vasculopathy with antimyelin oligodendrocyte glycoprotein antibodies in COVID-19

**DOI:** 10.1212/NXI.0000000000000813

**Published:** 2020-06-10

**Authors:** Ashwin A. Pinto, Liam S. Carroll, Vijay Nar, Aravinthan Varatharaj, Ian Galea

**Affiliations:** From the Wessex Neurological Centre (A.A.P., L.S.C., V.N., A.V., I.G.), Southampton General Hospital, Southampton, UK; and Clinical Neurosciences (A.A.P., A.V., I.G.), Clinical and Experimental Sciences, Faculty of Medicine, University of Southampton, UK.

A 44-year-old right-handed woman reported a gradual onset of right hand incoordination seven days after the onset of minor respiratory symptoms and pruritus due to COVID-19 infection. Over 48 hours, the patient developed word-finding difficulties and progression in right arm weakness leading to presentation to the emergency department as a suspected stroke.

Neurologic examination confirmed a mild expressive and receptive dysphasia, visual and sensory inattention, and Medical Research Council grade 4/5 weakness in the right arm and right leg. There was a rash on the chest wall bilaterally but no abnormal respiratory findings.

Blood workup confirmed normal results for full blood count (lymphocytes 1.5 × 10^9^/L), C-reactive protein, lactate dehydrogenase, and ferritin. In addition, the blood tests for antinuclear antibody, antineutrophil cytoplasmic antibody, anticardiolipin immunoglobulin G and immunoglobulin M, lupus anticoagulant and cold agglutinins were negative. The HIV and syphilis serologies were also negative. Severe acute respiratory syndrome coronavirus 2 (SARS-CoV-2) PCR from nasopharyngeal swab was positive.

MRI of the head with gadolinium and magnetic resonance angiography at presentation showed T2-hyperintensity within the centrum semiovale bilaterally in a periventricular location, extending along the left temporal and occipital horns and into the subcortical deep white matter bilaterally, more extensive in the left hemisphere. There was perivascular enhancement within the lesions, although no diffusion restriction, hemorrhage, or mass effect was found, and magnetic resonance angiography was normal (figure, A and D). MRI scan of the spinal cord was unremarkable with no radiologic signs of myelitis. CT of the chest, abdomen, and pelvis was normal with no evidence of pulmonary COVID-19 involvement.

**Figure:**
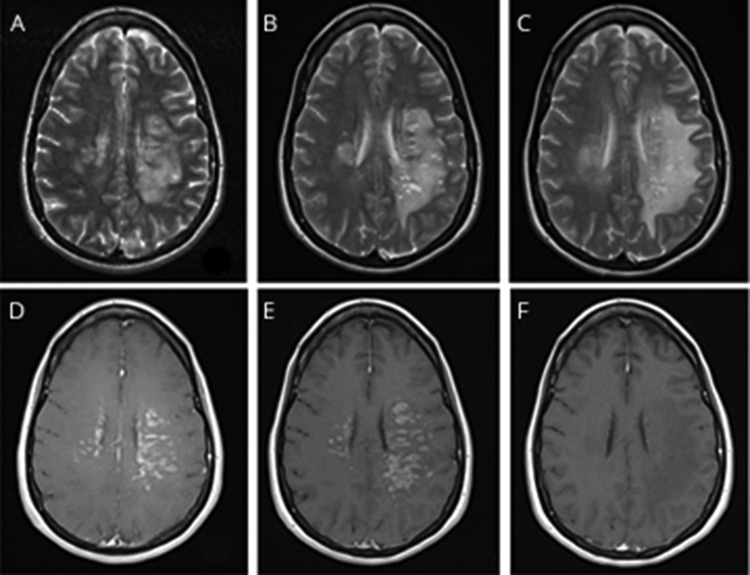
MRI appearances of CNS inflammatory vasculopathy with antimyelin oligodendrocyte glycoprotein antibodies in COVID-19 T2-weighted axial images at day 1 (A), day 6 (B), and post-treatment day 17 (C). Postcontrast axial images at day 1 (D), day 6 (E), and post-treatment day 17 (F).

CSF analysis showed 13/mm^3^ white cells (all mononuclear), red cells <1, protein 507 mg/L, glucose 2.9 mmol/L (serum glucose 6.3 mmol/L) with negative PCR for SARS-CoV-2, herpes simplex types 1 and 2, and JC virus. Oligoclonal bands were absent in the CSF. Although of potential relevance, serum and CSF cytokine analysis was unavailable for practical reasons during the university restrictions on activity in the laboratory.

There was clinical deterioration over the next 6 days with the development of severe aphasia and no antigravity movements of the right upper limb or at the right hip and knee. Repeat MRI brain scan, 6 days after presentation, showed progression of the bilateral centrum semiovale and white matter changes with extension into both hemispheres and more pronounced perivascular enhancement (figure, B and E). There were multiple, new cystic spaces without CSF signal consistent with enlarged perivascular spaces. Repeat CSF analysis on day 6 showed 8 mononuclear cells only and negative repeat SARS-CoV-2 PCR.

Treatment was initiated at day 6 with IV methylprednisolone (IVMP) 1 g daily for 5 consecutive days, followed by oral prednisolone 60 mg daily. The patient did not receive experimental antiviral treatment of COVID-19. On day 8, plasma exchange (PLEX) at 3.5L/d (1.5 plasma volumes) was commenced. There was a rapid clinical improvement in the neurologic deficit after the patient started immunomodulatory treatment. The patient had normal speech, almost full power in the right arm and leg, and no visual or sensory inattention at day 18 after 5 sessions of plasma exchange and IVMP. The MRI of the brain scan, after PLEX treatment on day 17 (figure, C and F), showed residual white matter vasogenic edema but no evidence of residual perivascular contrast-enhanced changes. Two weeks after discharge from hospital, an antimyelin oligodendrocyte glycoprotein (MOG) antibody test requested on admission was reported as positive.

Several classic autoimmune neurologic sequelae following COVID-19 have been described to date.^1^ However, this case was unusual for classic anti-MOG disease for a number of reasons. When solitary brain involvement occurs in the absence of opticospinal disease, the clinical and radiologic presentation is usually similar to that of acute disseminated encephalomyelitis,^2^ unlike here. In addition, perivascular enhancement is exceedingly rare in anti-MOG syndromes,^3^ with only one case reported.^4^

We hypothesize that a parainfectious anti-MOG antibody response combined with endothelial dysfunction to cause this unique clinicoradiologic CNS presentation. Vascular complications are increasingly recognized in COVID-19. The angiotensin-converting enzyme 2 receptors targeted by SARS-CoV-2 are expressed by endothelial cells in multiple organs including the brain.^5^ Recent histopathology from patients with COVID-19 has demonstrated a lymphocytic endotheliitis in the lungs, heart, kidney, small intestine, and liver with evidence of infarction.^6^ The blood-brain barrier breakdown secondary to endotheliitis, as suggested by the linear and punctate enhancement, may have facilitated the entry of anti-MOG antibodies to initiate the disease process and resulted in the unusual clinical and radiologic picture. The enlarged perivascular spaces returned signal higher than the CSF on fluid-attenuated inversion recovery sequences, which may represent distension by leucocytes migrating across the cerebral endothelium before traversing the glia limitans.^7^ The twice negative CSF SARS-CoV-2 PCR supports the idea that the CNS pathology was not because of parenchymal infection. The response to IVMP and PLEX was striking and is in keeping with the hypothesis of an immune-mediated process.
